# Isomorphic decisional biases across perceptual tasks

**DOI:** 10.1371/journal.pone.0245890

**Published:** 2021-01-22

**Authors:** Mario Treviño, Santiago Castiello, Oscar Arias-Carrión, Braniff De la Torre-Valdovinos, Ricardo Medina Coss y León

**Affiliations:** 1 Laboratorio de Plasticidad Cortical y Aprendizaje Perceptual, Instituto de Neurociencias, Universidad de Guadalajara, Guadalajara, Jalisco, México; 2 Department of Experimental Psychology, University of Oxford, Oxford, United Kingdom; 3 Unidad de Trastornos del Movimiento y Sueño, Hospital General Dr. Manuel Gea González, Ciudad de México, México; 4 Centro Universitario de Ciencias Exactas e Ingenierías, Universidad de Guadalajara, Guadalajara, Jalisco, México; Johns Hopkins University, UNITED STATES

## Abstract

Humans adjust their behavioral strategies to maximize rewards. However, in the laboratory, human decisional biases exist and persist in two alternative tasks, even when this behavior leads to a loss in utilities. Such biases constitute the tendency to choose one action over others and emerge from a combination of external and internal factors that are specific for each individual. Here, we explored the idea that internally-mediated decisional biases should stably occur and, hence, be reflected across multiple behavioral tasks. Our experimental results confirm this notion and illustrate how participants exhibited similar choice biases across days and tasks. Moreover, we show how side-choice behavior in a two alternative choice task served to identify participants, suggesting that individual traits could underlie these choice biases. The tasks and analytic tools developed for this study should become instrumental in exploring the interaction between internal and external factors that contribute to decisional biases. They could also serve to detect psychopathologies that involve aberrant levels of choice variability.

## Introduction

Humans are generally thought to maximize expected utilities by making choices that render the highest payoffs [1–3]. They achieve this by continuously adjusting their choice variability, which can be intentionally increased/decreased in search for actions that yield more rewards [4]. However, most people also exhibit systematic choice biases that lead to sub-optimal outcomes [5–8]. Such biases constitute the tendency to choose one action over others, with low variability from trial to trial (*i*.*e*., it is inversely related to adaptivity [9]) and no apparent goal or function. At the extreme, high levels of choice stereotypy characterize some human psychopathologies and mental disorders [10]. Similar manifestations of sub-optimal choice behavior have also been documented for rats [11], mice [6, 12, 13], and pigeons [14].

Human choices are influenced by sensory features, contextual factors, and display substantial individual differences [15]. Although it is clear that humans employ adaptive mechanisms to cope with dynamic environments, they also typically exhibit plenty of behavioral attributes that are primarily innate and relatively stable through life [16–18]. In the laboratory, sensory (visual) and decisional (non-visual) biases can be studied and dissected by employing specific task instructions for a two-alternative forced-choice (2AFC) task with symmetric alternatives [7]. In the 2AFC paradigm, participants are 'forced' to choose one from two options based on a perceptual criterion. Similarly, decisional (*i*.*e*., non-perceptual) side-choice biases can be studied through unforced choice (2AUC) tasks with equally rewarded alternatives [13, 19, 20]. Thus, in the 2AUC approach, the discriminative stimulus does not predict reward. Interestingly, using the latter task, we have found that decisional biases were independent of stimulus discriminability and the predictive value of sensory stimuli. We also found that side-choice behavior varied widely across participants but was stable for each individual across experimental days, suggesting the existence of diverse task-solving strategies [13].

Decisional biases are not exclusive to psychophysics and value-decision procedures. Using a memory detection task, researchers have shown that such response preferences are also stable across time and experimental conditions [19, 21–25]. Therefore, an intriguing possibility is that at least a portion of the observed decisional biases could derive from internal variables that are entirely independent of the sensory features and/or the contextual factors involved. We thus hypothesized that if human participants stably exhibit side-choice biases, they should co-vary with other behavioral metrics when the same individuals solved unrelated tasks. Employing forced and unforced tasks [13], we confirmed that participants exhibited different but consistent proportions of side-choice biases (*i*.*e*., decisional biases) across experimental days. Moreover, we found that the unique profiles of choice-bias production allowed us to use this behavioral attribute to identify participants, suggesting that individual traits might underlie side-choice behavior.

It is well established that subjects adjust their behavior in particular ways even if they are challenged under the same environmental contingencies. These adaptations reflect individual differences [26]. Our experiments and results support this notion, revealing that behavioral metrics from a reversal-learning task (employing volatile contingencies) predicted side-choice bias and alternation probabilities from individuals. Altogether, our results confirm the notion that stable internal elements gated decisional preferences and behavioral attributes that individuals exhibited reliably across multiple tasks. Thus, we propose that establishing a framework that distinguishes the internal and external factors that influence decisional biases is fundamental to understand how 'normal' and 'abnormal' behaviors are organized. The tasks and methods that we developed for this study could help detect and classify psychopathologies that involve aberrant levels of choice stereotypy.

## Materials and methods

### Participants

We conducted experiments using three tasks in 113 healthy volunteers (63 women and 50 men; age range (yr.): 16–39, mode: 27). For all tasks, participants performed a warm-up phase until obtaining 20 consecutive correct trials. Most participants were right-handed (≥ 90%), with normal or corrected vision, and without detectable neurological disorders or history of drug abuse. The ethics committee of our institution approved all these procedures (ET092018-271; Instituto de Neurociencias, Universidad de Guadalajara, México). We obtained written consent from all participants and gave them written instructions about the tasks they performed in silence.

### Free side-choice task (task #1)

**Fig 1:**
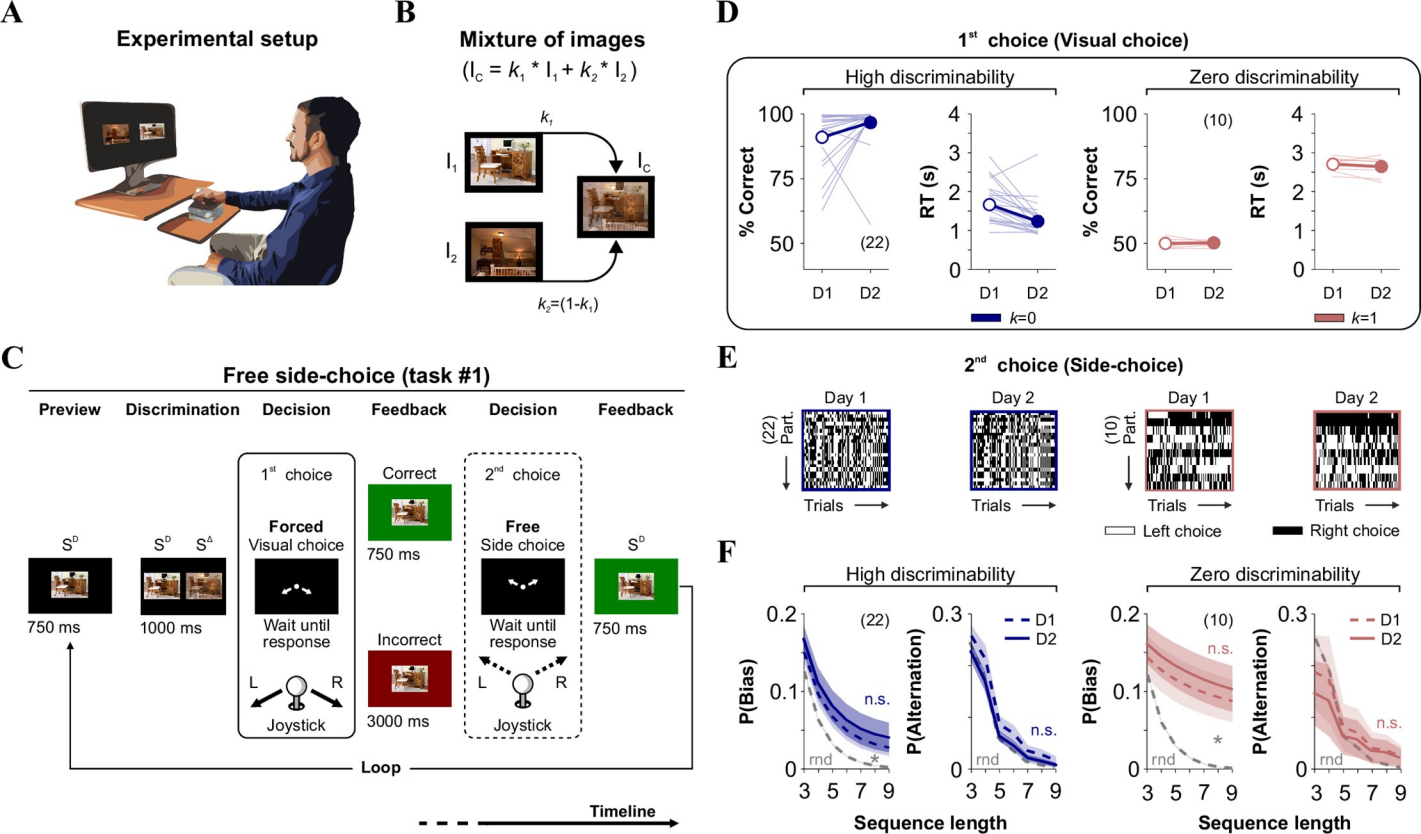
Stable side-biased and alternating probabilities in the free side-choice task. (A) Cartoon of the experimental setting where we measured perceptual and side-choices in humans. (B) The difficulty of discrimination is controlled by increasing image similarity (SIM, when *k* → 1). (C) Stimulus timeline of the visual task. Each trial involves a discriminative (visual) forced-choice (1^st^ choice, where the side of the S^D^ side predicts reduced delay interval), and a free side-choice (2^nd^ choice). We included auditory and visual feedback after recording joystick responses. (D) % Correct choices and response times (RT; in s) for two groups of participants tested with high (*k* = 0) or zero (*k* = 1) discriminability conditions. Thin lines: individual performances; thick lines and symbols: average ± S.E.M. (E) The side-choices from unsorted participants illustrated as colormaps with black (right) or white (left) rectangles across trials (x-axis). (F) Stable side and alternating choice probabilities across two experimental days. Number of subjects in parentheses.

To separately quantify visually guided choices and side-choice biases, we implemented a two-alternative forced-choice (2AFC) match-to-sample visual task (1^st^ forced visual choice) coupled with a second unforced-choice phase (2^nd^ free side-choice), as described in detail previously [13]. Briefly, participants sat upright in front of a computer monitor while we recorded their responses using a conventional joystick (Thrustmaster 2960623 USB Joystick; 1000 Hz) connected to a standard computer (Intel (R) Xeon (R) @ 3.40 GHz; 64 Bit Operating system; Graphics card: NVIDIA Quadro K600, 8 G.B.; Fig 1A). The visual stimuli (416 x 300 pixels; visual angle: 7° x 5°) were two pictures randomly positioned to the left and right sides of a projecting 27-inch computer screen (Dell P2414H, at a viewing distance of 60 cm). These two images had low semantic attributes, as validated previously [13]. We manipulated the task difficulty by creating distracter images (*i*.*e*., the S^Δ^, delta, or non-rewarded stimulus) with different similarities (SIM) relative to the sample stimulus (*i*.*e*., the S^D^, discriminative, or rewarded stimulus). We achieved this by using linear combinations of the two source images (Fig 1B). To solve the tasks, participants had to push the joystick in one direction specified by instructive arrows projected on the center of the screen. Choice regions occupied 5% of the overall search space. We measured the response times (RT, in s) as the interval between the visual stimuli' appearance and the moment when the participants placed the joystick in the appropriate response regions. After choosing, the participants had to let the joystick return to its baseline position. During the 1^st^ visual phase (2AFC), participants made their choice based on visual evidence by identifying the S^D^ location,^,^ which was projected for 1s to the left or right side of the S^Δ^ (the S^D^/ S^Δ^ positions were randomized). Next, two white arrows appeared on the center of the screen, pointing towards the right and left inferior corners, respectively, indicating the two response options (Fig 1C). These arrows remained on the screen until the participant responded. Next, the S^D^ was projected at the center of the screen for either 750 ms or 3 s if the participant answered correctly or incorrectly, respectively (*i*.*e*., a 1:4 relationship in the waiting intervals [27, 28]). During the 2^nd^ free side-choice phase (2AUC), the participants could visualize two white arrows that appeared on the center of the screen, pointing towards the right and left superior corners, indicating the two response options (Fig 1C). Here, participants could freely select upper left or right sides, respectively, with equal reinforcement for both sides. Thus, the choices during the second phase were used to characterize free side-choice behavior, as previously validated [13].

### Orientation discrimination task to measure sensory and decisional biases (task #2)

**Fig 2:**
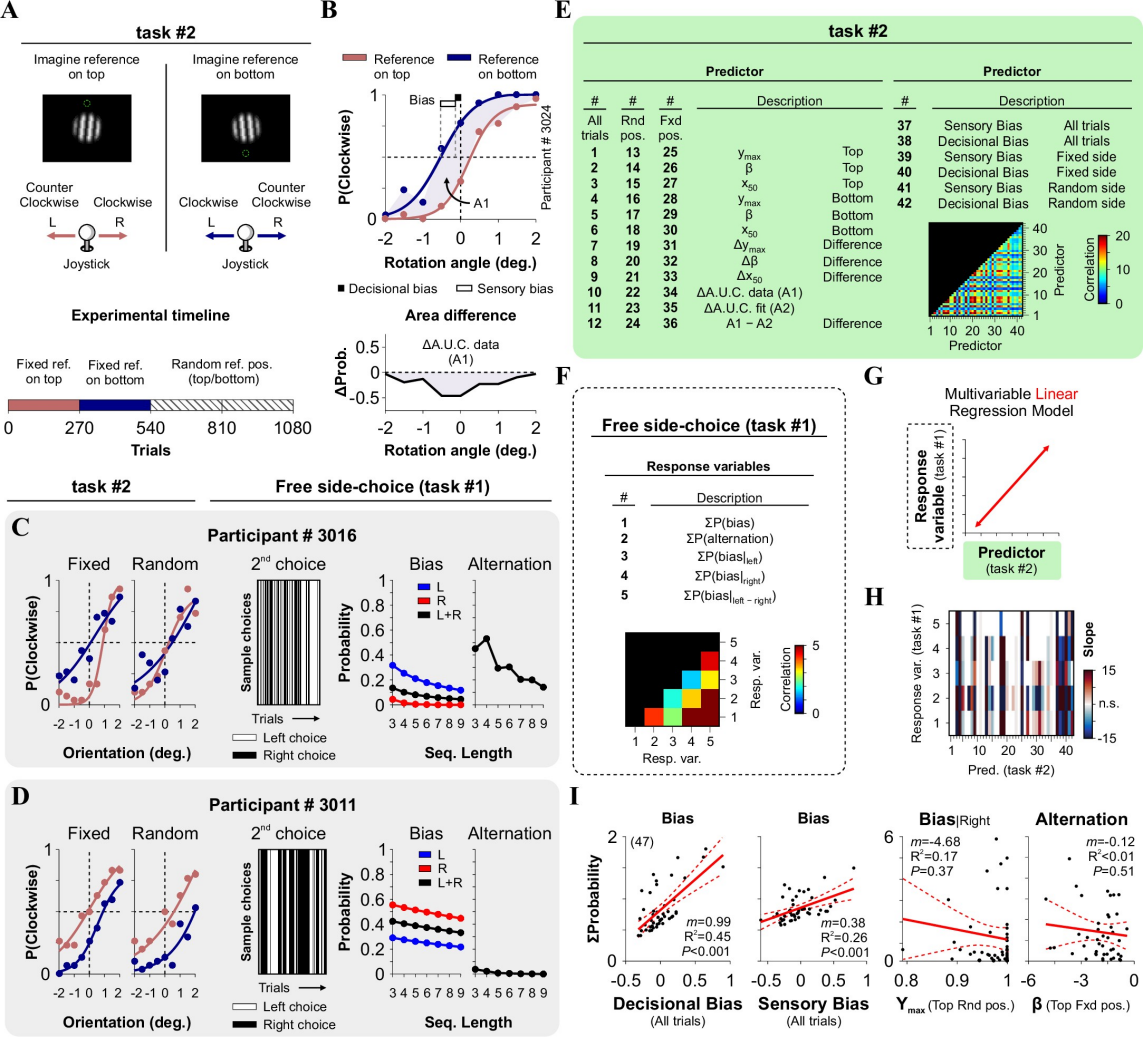
Comparison of choice-biases measured with two perceptual tasks. (A) Representation of López-Moliner’s task (task #2), which allows the dissection of sensory and decisional biases [7]. The diagram shows two trials in which the participants had to imagine a reference either on top (panel on the left) or on the bottom of the screen (panel on the right). (B) Example of an average choice response curves and psychometric curves from trials with top (indian red) and bottom (federal blue) references. Decisional (black square) and sensory (empty rectangle) biases can be extracted from each participant's psychometric fits. (C-D) Two examples of experimental results from participants #3016 and #3011 solving task #2 (left panels) and our free side-choice task (center and right panels). (E) List of the 42 predictors extracted from task #2. y_max_ (asymptote), β (steepness) and x_50_ (inflection point) correspond to the parameter fits from the psychometric curves. ΔA.U.C. (A1) is the area under the curve for the differences in choice responses with the reference on top and bottom (lower panel in B), and ΔA.U.C. (A2) corresponds to the differences from the psychometric fits obtained with the reference on top and bottom. (F) List of 5 response variables from the free side-choice task (task 1). Insets in panels E and F show the cross-correlation coefficients across predictors and response variables. Using PCA: the first 15 principal components explained 99.5% of the variance of the 42 predictors, whereas the first two principal components explained 90.74% of the variance of the 5 response variables. (G) Observed slopes from the MLRM comparing how parameters extracted from task #2 (rows) predicted measures from our free side-choice task (columns, task #1). Non-significant interactions appear as empty rectangles. (H) Sample linear regressions using relevant pairs of predictors/responses. Number of subjects in parenthesis.

To dissociate the contribution of sensory and decisional processes to global choice biases, we adapted a method developed by López-Moliner’s group [7]. This task consisted of determining whether a Gabor patch (projected for 100 ms) was rotated clockwise or counterclockwise relative to an imaginary reference placed either on the top or the bottom of the screen (green circle in Fig 2A). This task's premise is that sensory (visual) and decisional (non-visual) biases contribute distinctively to choice biases. More specifically, visual biases lead to different horizontal shifts in the psychometric curves depending on where the reference is placed (top or bottom), whereas decisional biases shift both curves towards a preferred side, independently of where the reference is placed (Fig 2B). The orientation of the Gabor patch was randomly selected from an angular range of ±2°, with 0.5° increments. To complete the task, the participants had to perform four blocks of 270 trials each. During the first two blocks, they were asked to imagine the reference on the same location for all trials (either up or down). During the last two blocks, the instructions randomly changed the location for the imaginary reference on every trial (experimental timeline in the lower panel from Fig 2A).

### Reversal learning task (task #3)

**Fig 3:**
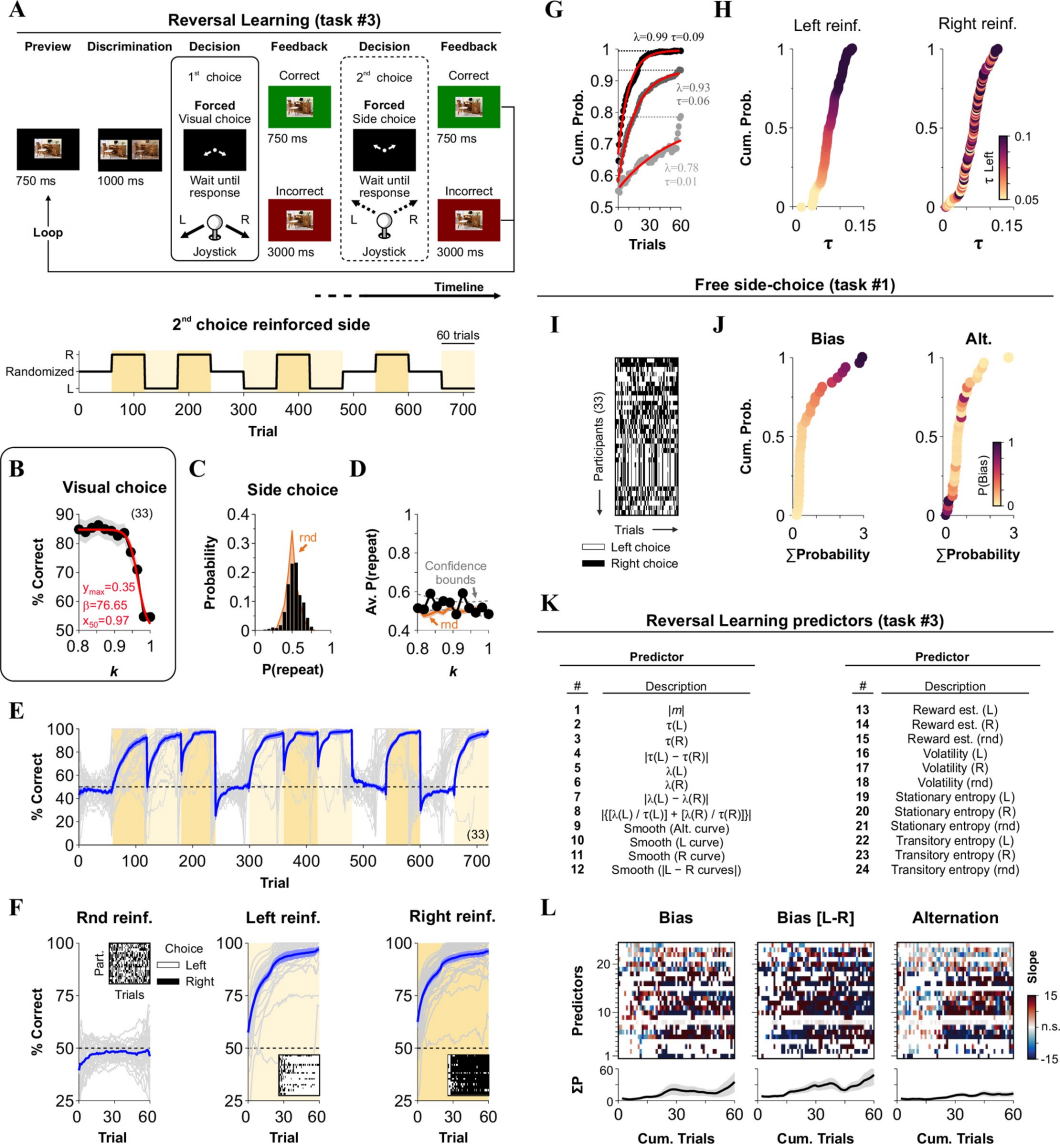
Comparing behavioral metrics from reversal learning with choice-biases from our free side-choice task. (A) Stimulus timeline of the reversal learning task (task 3), which consisted of a visual discriminative choice (1^st^ choice) followed by a forced side-choice (2^nd^ choice) during which we reinforced either left, right or alternating side-choices. (B) Average psychometric curve as a function of stimulus similarity (1^st^ choice). (C) Probability that side-choices involved the same side as the visual choice (*P*(repeat side)). Orange distribution corresponds to a random process derived from a binomial distribution (same number of trials). (D) Average %correct side-choice (2^nd^ choice) is fully independent of stimulus discriminability during visual choice. (E) Average %correct side-choice (2^nd^ forced side-choice, blue line) while using alternating or stationary left (vanilla yellow) or right (flax yellow) rewarding schedules. (F) Averaged learning curves are associated with the three reinforcement scenarios. Insets show sample side-choice colormaps from one of the four blocks for each reinforced contingency. (G) Sample mono-exponential fits to the learning curves from three participants. Taus (τ) and peak performance (λ) are extracted from these fits. (H) Cumulative probability distributions for the taus for left and right reinforcement. Participants are color-coded relative to τ_left_. (I) Side-choice colormaps for the same participants solving the free side-choice task (task #1). (J) Cumulative probability distributions for Σ*P*(Bias) and Σ*P*(alternation). Participants are color-coded relative to Σ*P*(Bias). (K) List of the 24 predictors extracted from task #3. The response variables from task #1 are identical to those presented in panel E from the previous figure. (L) Slopes for the MLRM comparing how 24 predictors from the reversal-learning task (task #3) predict, as a function of cumulative trials within each block, the three main response variables from the free side-choice task (labeled on top of each panel). Empty rectangles represent non-significant (n.s.) slopes. Number of subjects in parenthesis.

To investigate the adaptability of participants to solve a task with changing demands, we used the same core elements of task #1, but modified the 2^nd^ choice phase to reinforce either variable side-choices (using randomly permuted but balanced reinforced locations [29]) or choices towards the left or right sides only (*i*.*e*., the task became a 2AFC). Reinforcement was achieved by using a 1:4 relationship in the waiting intervals for correct and incorrect responses [13]. We employed stimuli with variable discriminability during the 1^st^ choice (Fig 3B), and we switched reinforced contingencies for the 2^nd^ choice every 60 trials, for a total of 12 blocks/day. To project the visual stimuli for all tasks, we used programs written in MATLAB R2016a (MathWorks, Inc.; Natick, USA) using the Psychophysics Toolbox extensions (PTB-3).

### Analysis of choice behavior

Participants selected left or right sides during the 2^nd^ side-choice phase of our experiments (task #1 and task #3). They typically exhibited a choice bias towards a preferred side [6, 13]. To illustrate participants' side-choices, we employed colormaps to plot the trials with the left or right responses (from the 2^nd^ choice) as white or black rectangles, respectively. Next, to quantify such side preferences, we measured the probability that sequences of side-choices were produced towards the same side or in alternation. These probabilities were quantified by using a pair-wise alignment method that consisted of sliding a query sequence of side-choices along with the choice records from each participant. A sequence was considered to be present in the choice record when the alignment rendered a perfect match for the entire length of the query sequence (*i*.*e*., observed choices = query sequence; sequence length refers to the number of trials of the sequence). The probability of occurrence of a choice sequence was then calculated by dividing the number of times that it was found in the choice record from each participant by the maximum number of times that it could fit within the total trials without interfering with any identical sequence that generated a count in previous trials, as explained and justified previously [6]. To complete this analysis: we added the counts of complementary sequences (side-biased: [‘LL…L’ + ‘RR…R’]; alternation: [‘LR…L’ + ‘RL…R’]) and normalized them to an equivalent number of training trials, thus allowing group comparisons. Note that measuring probabilities instead of the number of cases was crucial because the alignment method implicitly increases the counts of shorter sequences contained in longer ones [6, 30]. As a reference, we also created randomized choice patterns (rnd, binomial distribution; gray dotted line in Fig 1F) of equal length to the original choice records and measured biased and alternating sequences' probabilities.

To analyze the experimental data from task #2 [7], we first quantified the probability of estimating a clockwise rotation of the patch (*P*(*Clockwise*)) as a function of the rotation angle for trials performed when the imaginary reference was placed on top (reference on top) or bottom (reference on bottom) of the screen, respectively (Fig 2A and 2B). Two psychometric curves/participant (one for each reference) were then estimated by fitting the following logistic function (using 'lsqcurvefit' from MATLAB):

$$P(Clockwise)=\frac{y_{max}}{1+e^{-\beta (x-x_{50})}}$$ (1)

where *P(Clockwise)* is the probability of estimating a clockwise rotation of the patch at *x*°, *y*_*max*_ is the curve´s maximum value, *e* is the natural logarithm base (Euler’s number), *β* is the slope (proportional to choice variability [9]), and *x*_*50*_ is the *x*-value of the sigmoid's midpoint (Fig 2B). The magnitude of the decisional bias (*i*.*e*., non-visual component) for each participant was estimated as the midpoint between *x*_*0*_|_ref.top_ and *x*_*0*_|_ref.bottom_. The magnitude of the sensory bias (*i*.*e*., visual component) was taken as the absolute difference between any *x*_*0*_ and the decisional bias (*i*.*e*., this value is spatially invariant; Fig 2B). As a secondary estimate of the sensory bias, we calculated the area under the curve of the difference between the two psychometric curves derived either from raw data (A1), or from the fitted logistic curves (A2) *P(Clockwise)*| _ref.top_*—P(Clockwise)*| _ref.bottom_ (ΔA.U.C.; Fig 2E). We incorporated both measurements in our table of predictors because each approach has a distinctive strength: i) the difference calculated from the raw data corresponds to a clean and unprocessed estimation, whereas ii) the difference extracted from the fitted sigmoids is less sensitive to outliers.

For the experiments involving task #3 (illustrated in Fig 3), we reinforced single-side (Left reinf. or Right reinf.) or variable responses (rnd reinf.) using a training paradigm with contingencies that favored side-choices (left or right) or variable/random choices, respectively. For each condition, we took the block-averaged choice records from each participant (*y*) and fitted them with mono-exponential curves with the form:

$$y=\lambda -(y_{0}∙e^{-\tau x})$$ (2)

where *λ* corresponds to the maximum average performance achieved during the block, *y*_*0*_ is the initial % Correct responses (a free parameter in the fitting procedure), *x* are the trials within the block, and *τ* is the rise constant.

We used a Bayesian approach to estimate reward (*r*) and volatility (*v*) probabilities given the history of trial outcomes (rewards) for each participant, as described in detail previously [31]. The Bayesian learner (BL) is a hierarchical generative model that estimates an agent’s beliefs about the probability of reward (*r*) at every trial. Thus, from the BL perspective, the participant's goal is to estimate *r*_i+1_ from all previous data (*r*_≤i_) to increase the chances of obtaining a reward at the subsequent trial. Rewards in task #3 involved waiting for a shorter interval combined with a green feedback screen (Fig 3A). Using a Markovian setting, the change of *r* was represented with a beta distribution:

$$P\left(r_{i+1}|r_{i},v\right)∼\beta \left(r_{i},V\right)$$ (3)

where *r*_i_ defines the mean of the distribution, and *V* = exp(*v*) defines the distribution's width (*i*.*e*., the integrals were performed in log space; see below). *V* corresponds to the volatility as it controls how the observed outcomes of decisions influence the estimated reward probability between trials. Therefore, if the environment goes through stable and volatile phases, the subject must adapt to this changing volatility to perform optimally. For simplicity, the change of *v* was represented with a Gaussian distribution:

$$P\left(v_{i+1}|v_{i},k\right)∼N\left(v_{i},K\right)$$ (4)

where *K* = exp(*k*) controls the rate of change of volatility. We mapped *k* values from log(5e-4) to log(20) with 0.2 irements for our analysis. A small *k* value leads to a narrow transitional distribution (small changes in *v*), whereas a large *k* value leads to a wide distribution (large changes in *v*).

In order to make a decision at trial i+1, the BL requires to estimate the reward rate *r*_i+1_. The BL must therefore compute the marginal distribution *P*(*r*_i+1_) from the joint probability distribution *P*(*r*_i+1_,*v*_i+1_,*k*) by integrating over *v*_i+1_ and *k*:

$$P(r_{i+1})=\iint P(r_{i+1},v_{i+1},k)dv_{i+1}dk$$ (5)

The current estimate of the reward rate was then given by the mean of this distribution:

$${\hat{r}}_{i+1}=\int r_{i+1}P(r_{i+1})dr_{i+1}$$ (6)

All integrals were performed numerically. The BL was applied to the data from each participant, and it returned *r* and *v* as a function of the experimental trials.

To indirectly assess the overall bias, we used the stationary (Shannon) entropy:

$$H(Response\;side)=-\sum_{i=1}^{2}P(s)*{log}_{2}[P(s)]$$ (7)

where *s* is the response side (left or right).

To measure the level of unpredictability of the transitions between consecutive choices [32], we measured the transition entropy for side-choices as:

$$H(Response\;side|prior\;response\;side)=-\sum_{i=1}^{2}P(s_{i})\sum_{j=1}^{2}P(s_{i}|s_{i-1})*{log}_{2}[P(s_{i}|s_{i-1})]$$ (8)

where *s*_*i*_ is the response side (left or right), and *s*_*i-1*_ is the prior response side (left or right). The entropy values ranged between 0 and 1.

Finally, to estimate the contributions of previous visual choices, side-choices, and their outcomes (success or failure) on the production of current side-choices, we carried out multiple linear regression (MLRM) analyses, as previously described [6, 13, 29].

### Statistical analysis

We used t-tests, parametric and non-parametric tests for statistical comparisons, and repeated measures ANOVA (RM-ANOVA) tests with Bonferroni’s or Wilcoxon Signed Rank *post hoc* tests for group comparisons. We compared the probability distributions using Kolmogorov-Smirnov (KS) tests. To determine the MLRM analyses' significance, we compared the actual regression coefficients against 1000 surrogate data sets generated by shuffling the predictors [33]. With this approach, we established the empirical significance of the observed coefficients by comparing them against coefficients obtained with randomly permuted predictors (surrogates) to test the null hypothesis that the regression coefficients might have been generated by chance. We also employed principal component analysis (PCA) to quantify the explained variance in subsets of predictors and response variables from task #2. The total variance corresponded to the sum of variances from all original components. The fraction of explained variance by a principal component (PC) was the ratio of that PC's variance and the total variance. We illustrate our group data as averages ± S.E.M. with a significance set at *P* ≤ 0.05. For the experiments shown in Fig 1, we discarded from the analysis a participant who exhibited chance performance under high discriminability conditions (*i*.*e*., distracted or with low commitment to the task). For the experiments shown in Fig 2, we discarded six participants because they exhibited low performance either in task #1 (% Correct with [*k* = 0] ≤ 60%) or in task #2 (*P*(+2°) ≤ 60%). The number of participants used for each task's analysis is illustrated inside parentheses in the corresponding figure panels.

## Results

### Stable production of decisional biases in a free side-choice task

Our first task allowed us to sequentially measure visual performance (two-alternative-forced choice [2AFC] task; visual choice), and side-choice biases (two-alternative-unforced/free choice [2AUC] task; side-choice) during consecutive phases within each trial (see Methods; Fig 1A–1C; [13]). For the 1^st^ visual choice (2AFC), participants had to identify which of the two stimuli was identical to a target stimulus (S^D^), by responding with a joystick down-left or down-right. We recorded the choices and response times (RT, in s) of the participants by connecting the joystick to a standard computer. Feedback for the visual task involved waiting longer times after incorrect choices (3000 ms) than after correct ones (750 ms; *i*.*e*., negative reward schedule; Fig 1C). Thus, the implicit motivation to solve the task was to discriminate correctly to finish the task promptly. During the 2^nd^ free side-choice (2AUC), participants responded either up-left or up-right to advance to the next trial, which involved visualizing the target stimulus for another 750 ms, regardless of the chosen side (Fig 1C).

Next, we explored the stability of visual and side-choice behavior along consecutive days using either high (*k* = 0) or zero (*k* = 1; identical images) discriminability conditions. We found similar (paired t-test, *k* = 0; *P* = 0.07; k = 1, *P* = 0.58) visual performance across two consecutive days for both groups of participants (*k* = 0; Choice | D1: 91.00% ± 2.04%; D2: 96.66% ± 1.94%; RT | D1: 1.67s ± 0.13s; D2: 1.24s ± 0.11s, *n* = 22; *k* = 1; Choice | D1: 49.97% ± 0.52%; D2: 50.24% ± 0.35%; RT | D1: 2.70s ± 0.05s; D2: 2.64s ± 0.07s, *n* = 10; Fig 1D). RTs were stable with zero discriminability (paired t-test, *P* = 0.20), and they were ~ 23% smaller in high discriminability conditions (paired t-test, *k* = 0; *P* < 0.001). To visualize the side-choices of the participants (2^nd^ choice from task#1), we employed colormaps to illustrate left and right choices with white or black rectangles, respectively (Fig 1E). We then measured the probability of producing choice sequences towards the same side (*P*(Bias) = *P*(Bias|_left_) + (*P*(Bias|_right_)), or in alternation (*P*(Alternation); see Methods) [6, 13]. We found remarkably similar side-choice and alternation probabilities across consecutive days (*k* = 0; Prob. Bias | Repeated Measures [RM] ANOVA test, *F* = 0.49, *P* = 0.87; Prob. Alternation | Repeated Measures [RM] ANOVA test, *F* = 1.13, *P* = 0.29; *k* = 1; Prob. Bias | Repeated Measures [RM] ANOVA test, *F* = 0.13, *P* = 0.96; Prob. Alternation | Repeated Measures [RM] ANOVA test, *F* = 0.22, *P* = 0.92, Fig 1F). Using a paired t-test, we confirmed that the stability of this measurements held at the individual level (Prob. Bias | *k* = 0, *P* = 0.13; *k* = 1, *P* = 0.10; Prob. Alternation | *k* = 0, *P* = 0.35; *k* = 1, *P* = 0.08). As a reference, we calculated the probability distributions for a random binomial process (rnd, gray dotted lines in Fig 1F). Note how *P*(Bias) increased it's overall difference relative to *P*(rnd) when using zero discriminability. These results illustrate how human side-choice behavior is stable over time. Intra-individual (temporal) stability in free side-choice behavior was present in both groups irrespectively of the discriminability conditions.

### Decisional biases measured across different perceptual tasks

We next wondered whether participants’ side-choices would manifest similarly across different tasks. For that, in a new group of naïve participants, we conducted the free side-choice task (task #1; *k* = 1) followed by a second orientation discrimination task (task #2, Fig 2A)[7]. We illustrate sample results from participant #3024 solving task #2 in Fig 2B, with fitted psychometric curves to the observed averaged choices (Fixed side condition: Bottom | y_max_ = 1.00, β = -2.24, x_50_ = -0.52, A.U.C. = 3.26; Top | y_max_ = 0.92, β = -2.95, x_50_ = 0.21, A.U.C. = 5.15, Δ[A.U.C.] = -1.89; Sensory bias = -0.31; Decisional bias = -0.29; y_max_: asymptote, β: steepness, x_50_: inflection point).

Next, we compared the choice bias estimations from the 47 participants solving the orientation discrimination task (task #2) with their side-choice behavior extracted using the original free side-choice task (task #1). In Fig 2C and 2D, we illustrate examples from two participants solving both tasks. To assess a potential relationship between the behavioral metrics from these two tasks, we extracted 42 predictors from task #2 (Fig 2E), and five response variables from task #1 (Fig 2F). Notably, there were strong cross-correlations across some of these variables, suggesting that many of these parameters were interdependent of influenced by a common source (insets in panels from Fig 2E and 2F).

We then applied a multivariable linear regression model (MLRM) to predict the response variables from task #1 (Fig 2F and 2G). The MLRM regression coefficients revealed significant interactions between predictors and responses (warm and cold colors show significant positive and negative slopes, respectively, whereas a white color indicates a non-significant ‘n.s.’ slope against 1000 shuffles, Fig 2H**)**. In particular, predictors 37–42 from task #2, which directly quantified sensory and decisional biases, had strong and significant interactions with practically all response variables from task #1. This reveals a direct link between the decisional and sensory biases measured in task #2 and the side-choice bias and alternation probabilities extracted from task #1. We illustrate four relevant sample pair-wise linear regressions in Fig 2I. Interestingly, choice-biases measured in the free side-choice task (task #1 [13]) were better coupled to decisional than to sensory biases from task #2 (Fig 2I; [7]). These results confirm the idea that human choice biases are exhibited reliably across different tasks, possibly reflecting behavioral outputs with a stable and common source.

### Behavioral attributes extracted from a reversal-learning task predict decisional biases

Reinforced sides in 2AFC tasks are generally distributed in a randomized fashion so that decisional biases lead to chance performance [29]. Yet, we know that decision-makers can quickly adapt their behavior to contingencies that favor one over another option [10, 13]. We implemented a reversal-learning task (task #3) to measure such adaptations. To test visual discrimination capacities during the first visual choice, we modified task #1 by incorporating stimuli with 12 similarities (SIM) ranging from *k* = 0.8 to *k* = 1. We also changed the 2^nd^ side-choice phase to a negatively reinforced schedule to favor particular sides during training (in this context, choice biases corresponded to inverse adaptivity [9]; see Methods). We used the same feedback rules for both discriminative and reinforced side-choices (Fig 3A).

With this task #3, we trained 33 participants across 12 blocks of 60 trials each. For each block, we used variable or stationary rewarding epochs by reinforcing either randomized (rnd, randomly permuted balanced sides), or all-left (L), or all-right (R) sequences, respectively. We repeated each of these blocks four times so that all participants experienced all combinations of possible transitions across conditions (experimental timeline in the lower panel from Fig 3A).

As expected, visual choices depended on stimulus similarity (*F* = 19.3, *P* < 0.001), with performance dropping when *k* → 1 (logistic fit: y_max_ = 0.35, β = 76.65, x_50_ = 0.97; Fig 3B). We next measured sequential effects (*i*.*e*., 1^st^ choice influencing the 2^nd^ choice) by calculating the probability that the side-choice (2^nd^ choice) involved repeating the same side as with the visual choice (1^st^ choice). Interestingly, in these conditions, we found no apparent sequential effects between choices (*F* = 3.69, *P* = 0.08), with the observed side-choices following a random binomial process (rnd, orange; *F* = 0.37, *P* = 0.54; Fig 3C), and fully independent of stimulus similarity (*F* = 1.12, *P* = 0.31; Fig 3D). These observations reflect the lack of sequential effects or that such effects canceled each other out due to our balanced experimental design. Next, we calculated a moving average of the participants’ %correct choices during the 2^nd^ forced side-choice phase (blue line corresponds to group averaged data, Fig 3E). We separated the learning curves by reinforcement conditions (average of the last 10 trials within each block; randomized | 48.07% ± 1.39%; left | 96.03% ± 1.73%; right | 95.46% ± 1.49%; non-parametric Kruskal-Wallis test with Bonferroni correction, *P* < 0.001; *n* = 33; Fig 3F). The chance performance level (average from the four blocks with the same contingency) observed in the randomized-side reinforced condition (Rnd reinf.) implies that participants increased their choice variability during these blocks. We then took the block-averaged choice records from each participant and fitted them with mono-exponential curves to extract rising taus (τ) and peak values achieved (λ; three different examples in Fig 3G). The sorted τ values for left and right reinforcements covered a wide range from a τ_min_ ≈ 0.01 to τ_max_ ≈ 0.13 (Fig 3H).

To estimate the volatility (*v*) and reward (*r*) rates, we fitted an optimal Bayesian Learner (BL) model [31] to the side-choice records from the participants (not illustrated; see Methods). The hierarchical aspect of this model is that the change in *r* depends on *v*, and will change from trial to trial. For example, if a current trial is preceded by many trials with incorrect responses, then the model will estimate a high *v*, which will increase the change in *r*. In contrast, lower *v* values will lead to more stable *r* probabilities. Thus, when *r* is stable, then *v* will be small, which in turn implies that *r* will not get much influence by previous trials. Volatilities were similar with left and right reinforcements (randomized | 22.77 ± 0.51; left | 16.58 ± 0.40; right | 16.98 ± 0.42; *n* = 33), but they were ~35% higher with randomized reinforcement (non-parametric Kruskal-Wallis test with Bonferroni correction, *P* < 0.001). Likewise, the estimated reward rates from the BL were similar with left and right reinforcement (randomized | 48.85% ± 0.76%; left | 87.90% ± 1.70%; right | 87.14% ± 1.50%; *n* = 33), but were ~43% smaller with randomized reinforcement (*F* = 60.97, *P* < 0.001). Interestingly, participants with higher volatility exhibited a reduced performance (area under the curve) during the single side-reinforced (R^2^ = 0.75, *m* = -3.56, *P* < 0.001), but not during the randomized-side reinforced blocks (R^2^ < 0.001, *m* = 0.02, *P* = 0.91).

As secondary metrics to detect and quantify overall choice biases, we included the stationary (*i*.*e*., Shannon) and transition (*i*.*e*., conditional) entropies [32] (not illustrated; see Methods). In addition, we characterized the bias and alternation probabilities for the same participants when solving task #1 (Fig 3I and 3J). We then collected 24 predictors from task #3 (enlisted in Fig 3K) and applied an MLRM to predict the five response variables from task #1 (same response variables as those depicted in Fig 2F). The MLRM coefficients (plotted as a function of the training trial within each block) revealed significant interactions between predictors and responses (colored squares in Fig 3L; see Methods). These results demonstrate that choice biases and other behavioral attributes extracted from task #3 displayed a strong co-variation with metrics from task #1.

### Choice biases as an individuality measure

**Fig 4:**
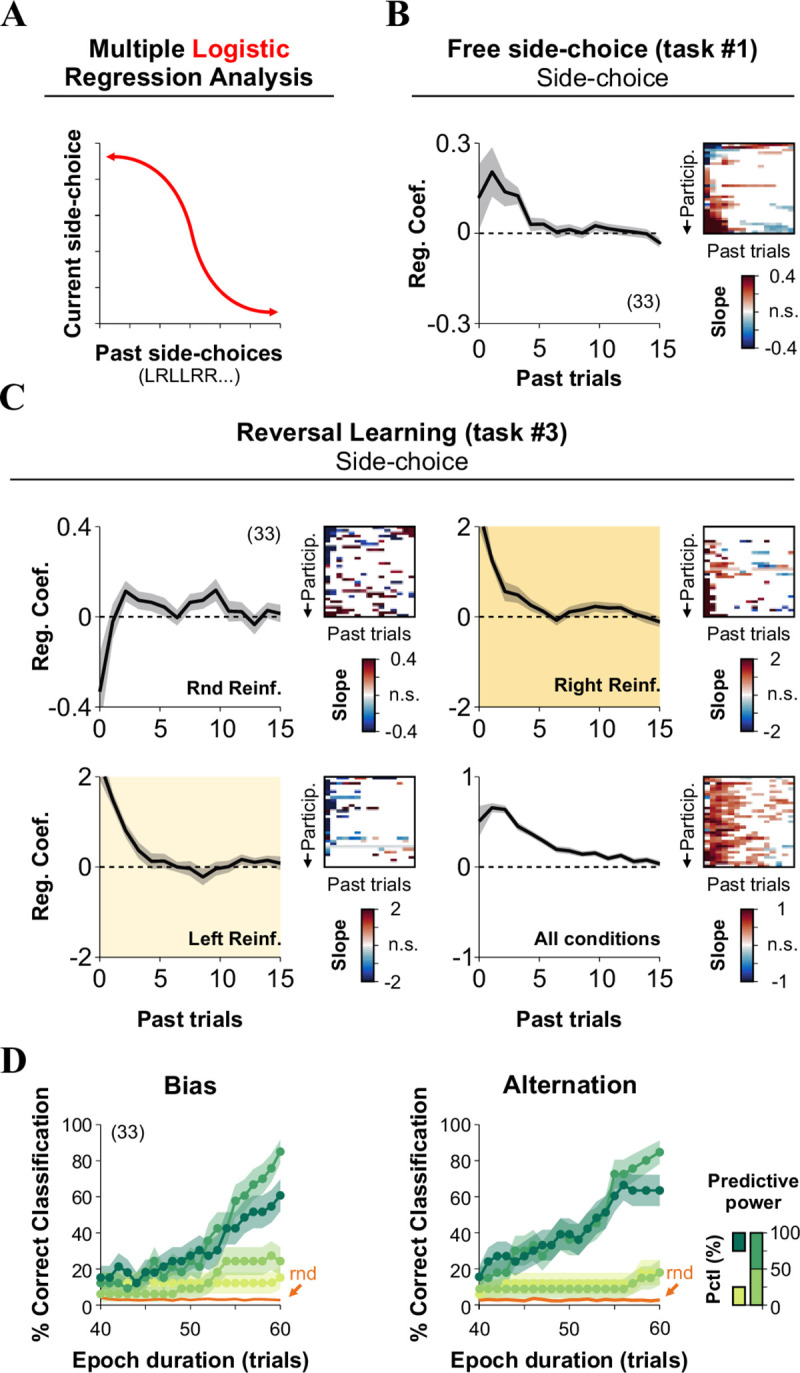
Past decisions influence side-choices in multiple tasks. (A) We conducted a multiple logistic regression analysis to predict current side-choice behavior from past side-choices, using the same data as in the previous experiment where participants solved the free side-choice (task #1, B) and the reversal learning (task #3, C) tasks. For task #1, the probability of repeating a side was strongly influenced by the amount of previous side-biased choices (B). For task #3, the odds of repeating the same side increased when side-choices were reinforced but decreased when alternating choices were reinforced (C). (D) Cumulative match characteristic curves as a function of epoch duration (x-axis). The curves were calculated by using different sets of predictors with their strength identified by using a stepwise regression learner. Classifications were performed using predictors falling in different percentile ranges, as indicated by the color bar on the right. Number of subjects in parenthesis.

Behavioral responses primarily depend on sensory cues, but they are also influenced by past choices and reinforcers [7]. To assess the dependence of current side-choices on previous choices, we applied a multiple logistic regression analysis (MLRA with a “logit” link; Fig 4A; [6]) to the behavioral responses from our last experiments involving task #1 and task #3. We calculated the regression coefficients (β) by using 15 previous trials [6, 29]. As expected, previous-side choices from both task #1 (Fig 4B) and task #3 (Fig 4C) had a strong influence on current side-choice behavior that outlasted at least 4–5 previous trials. The positive coefficients reflect increased odds of repeating the same side on the subsequent trials, whereas negative coefficients indicate alternation.

Because the MLRM from the previous section revealed that behavioral metrics extracted from multiple tasks strongly co-varied, we explored the possibility of using the predictors to identify the participants. We implemented a simple linear classifier that minimized the Euclidean distance between observed (*i*.*e*., the value of the predictor at a given trial within the block) and 'final' predictors extracted from the entire 60 trials/block (*i*.*e*., the value of the predictor at the end of the block). We then calculated the cumulative match characteristic curves by using increasing epoch durations (the number of cumulative trials from which potential matches were calculated [13]). As expected, we found that prediction accuracy grew by increasing the epoch duration, compared against classifications made with 1000 random permutations of the predictors (rnd, in orange). Moreover, accuracy grew faster when using stronger predictors (dark and shamrock greens), particularly those extracted from alternation probabilities (Fig 4D). We ranked the predictive power of the predictors on response variables from task #1 by using a stepwise regression learning procedure (Matlab Regression Learner App; *P*(Bias) | five best predictors (increasing strength): 22, 12, 13, 3, 1; five worst predictors (increasing strength): 24, 17, 4, 7, 11; *P*(Alternation) | five best predictors: 3, 7, 18, 12, 24; five worst predictors: 20, 13, 19, 1, 11; these numeric IDs correspond to predictor numbers displayed in Fig 3K). Interestingly, this approach revealed that the transition entropy was the best predictor for alternating choices, but the worst one for biased-choices. In contrast, the rectified slope of the linear regression applied to the participants' side-choices in the randomized-side reinforced blocks was the best predictor for biased-choices but one of the worst for alternating choices. These results confirm the idea that bias and alternation choice probabilities can be used to predict the identity of the participants.

## Discussion

We used three tasks aimed to explore perceptual and internally-guided choice biases [13]. The tasks were easy to perform, allowing us to test 1000 trials/day from each participant. The measured choice-biases showed heterogeneous magnitudes and preferred sides, indicating that they were not an artifact from our experimental apparatus and/or experimental conditions [6]. Furthermore, we found that each participant's side-choice behavior was remarkably stable across experimental days. Particularly under low discriminability conditions, such a stable representation was reflected as distinctive patterns of side-choice biases at the individual level. In contrast, side-choices (measured during the 2^n^d free side-choice phase) tended to be more similar across participants under high discriminability conditions (task #1). This was reflected as a strong group choice coherence and could be explained by at least two factors. The first one is that all participants were visually tested with precisely the same sequence of pseudo-randomized stimuli. The second one is that side-choices in task #1 exhibit strong sequential effects with respect to choices produced during the discriminative phase [13]. In agreement, such strong side-choice coherence was practically absent in the experiments performed with zero discriminability. The marked difference between the side-choice and chance level probability distributions make the experiments performed with zero discriminability a better approach to characterize side-choice behavior.

One of our main results is that behavioral attributes extracted from the participants solving the different tasks showed a strong intra-individual consistency that could not be explained by chance [21, 22, 34–36]. Furthermore, the strong MLRM regression coefficients between side-choices in task #1 and choice biases from task #2 suggests that decisional preferences were exhibited reliably across these tasks. An alternative way of explaining it is that the measured behavioral outputs were influenced by stable and shared sources.

In humans, evidence suggests that individual differences influence learning about the predictive value of multiple cues [37], and differences in the learning curves unveil distinctive adaptive strategies [38, 39]. In agreement, we found that side-choice biases could be used to identify participants that solved our tasks, in line with what has been shown for memory detection tasks [21, 22]. Therefore, an interesting possibility is that choice biases could reflect human psychological traits that extend to other decision-making settings. Furthermore, such decisional biases could substantially impact the perceptual evidence accumulation process, as understood and analyzed through sequential-sampling models [27].

By using reversing contingencies, we found that side and alternating choice sequences could be effectively reinforced. The premise here was that participants with more flexible choices had greater choice variability (lower bias) whereas those with less flexible choices had lower variance (higher bias)

In general, human decisions are adaptive and involve integrating uncertain information in different time scales [9, 13]. Thus, from this perspective, the learning curves from the participants solving task #3 could reflect a Bayesian inference strategy, with a strong influence of previous side-choices [13, 40, 41]. Indeed, the training protocol in this task involved adaptive side-choice behavior (*i*.*e*., action volatility), and reward estimations were beneficial to subjects to discount information with passing trials. Usually, volatility refers to blocks of trials in which the optimal solution is continually changing (*v*.*gr*., on a trial-by-trial basis). Volatility in positive reinforcement studies has been widely explored (*v*.*gr*., produce positive outcomes; [31, 42], as well as with negative reinforcement schedules (*v*.*gr*., avoid adverse outcomes; [43, 44]. Such a reversal-learning paradigm serves to measure the ability of participants to suppress previously learned responses. Thus, our task #3 provided a proper setting to test the trade-off between exploration (high choice variance) and exploitation (low choice variance). Indeed, it has been shown how individual differences in this learning process can reflect different implicit assumptions about sequence complexity, leading to performance trade-offs [9]. Interestingly, the learning curves quantified through this approach are thought to reflect the flexibility of responses, and changes in these learning parameters have been linked to impulsive and compulsive behaviors in a variety of psychopathologies [45, 46]. Therefore, the analysis of behavioral outputs in our multiple reversal-learning task (task #3) might detect such psychopathologies. Notably, previous evidence suggests that controlled motor variability could also be beneficial in some conditions [47, 48]. For example, task-relevant variability could explain some individual differences in learning rates and motor development [48, 49].

Altogether, our results illustrate how participants exhibited isomorphic side-choice biases, manifested reliably across days and tasks. Many other behavioral metrics co-varied across tasks and could be employed to predict the side-choice preferences that were not guided by any feedback. Also, it has been shown that when reinforcement of specific side and alternating choice sequences is removed, then participants tend to return to their 'innate' side choice behavior [13]. A parsimonious explanation is that all these behavioral metrics linked to decisional biases are influenced by a common, internal, and stable source. Our results support this notion, showing how human choice-biases can be taken as an intrinsic characteristic that varies distinctively among humans.

A complete picture of all the variables and mechanisms contributing to the manifestation of repetitive behaviors is still missing. However, the propensity to develop such choice stereotypies should also depend on genomic factors [50]. Structured behavioral patterns are essential for survival, and highly skilled behavior involves sequences of repetitive actions [51]. Response biases are also tightly correlated to inhibitory control [23] and could be linked to delayed discounting (*i*.*e*., the reduction in the value of an outcome when it's reception is delayed; [52]). Some stereotypies could also reflect reinforced habits that are quite persistent and hard to extinguish [51]. At the extreme, a high level of behavioral stereotypy could characterize some forms of impulsivities and psychopathologies [10, 21, 52]. For example, there is a parallelism between choice biases and the stereotypical clinical behavior exhibited in some cases of autism spectrum disorder (ASD; [53]. There is an ongoing debate about what maintains this type of behavior. One possibility is that repetitive actions produce constant and predictable self-sensory information [53], and given that people with ASD have an intolerance to uncertainty (IU; [54]), a stereotypical source of self-sensory information could reduce sensory uncertainty. Given that ASD has been characterized as a spectrum or a continuum [55], a proposal is that we could use the level of stereotypical side-choice biases from the presented tasks to predict severity within this range. Moreover, stereotypies might also be related to the production of non-goal-directed behaviors, which are impaired in schizophrenia [56], Tourette’s syndrome, and obsessive-compulsive disorder patients [57, 58]. Other psychopathologies, such as attention deficit hyperactivity disorder (ADHD), and Down’s Syndrome, show elevated amounts of intra-individual variability in basic motor skills [10]. Thus, it would be interesting, if not crucial, to explore how the 'behavioral fingerprint' extracted from the reversal-learning task predicts psychological traits and syndromes.

To summarize: we showed that human decisional biases were similar across tasks and days. Although choice stereotypy probably emerges under a variety of environmental conditions, we here described how this behavior could be precisely characterized in the laboratory using quantitative metrics. Finally, we showed how side-choice biases could be used as a ‘behavioral signature’ to identify participants.
